# Redox Dynamic Interactions of Arsenic(III) with Green Rust Sulfate in the Presence of Citrate

**DOI:** 10.1021/acs.estlett.4c00700

**Published:** 2024-10-15

**Authors:** Jagannath Biswakarma, Molly Matthews, James M. Byrne

**Affiliations:** † School of Earth Sciences, University of Bristol, Bristol BS8 1RJ, United Kingdom

**Keywords:** iron (oxyhydr)oxides, As(V), organic ligands, electron transfer, speciation change, phase transformation

## Abstract

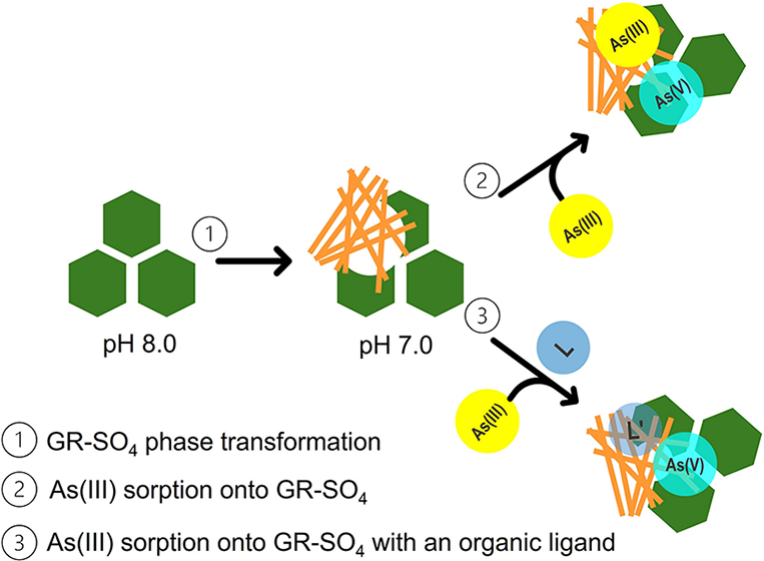

Arsenic is a global pollutant. Recent studies found that Fe(II) can oxidize As(III), but the extent of oxidation with mixed-valent iron minerals and the mechanisms involved are unknown. In this study, we investigated whether As(III) can be oxidized under reducing conditions using green rust sulfate (GR-SO_4_), an Fe mineral containing both Fe(II) and Fe(III). Batch sorption experiments showed that GR-SO_4_ (1 g L^–1^) effectively sorbs environmentally relevant concentrations of As(III) (50–500 μg L^–1^) under anoxic, neutral pH conditions with and without citrate (50 μM). X-ray absorption near-edge structure spectroscopy analysis at the As K-edge demonstrated that approximately 76% of As(III) was oxidized to As(V) by GR-SO_4_. Complete oxidation of As(III) was observed in the presence of citrate. As(III) oxidation can be linked to the phase transformation of GR-SO_4_ to goethite, resulting in new reactive Fe(III) species that plausibly drive oxidation. Citrate enhanced this process by stabilizing Fe on the mixed GR-SO_4_/goethite surface, preventing its reduction back to Fe(II) and facilitating further As(III) oxidation without significant Fe loss to the solution. This study highlights the cryptic As(III) oxidation that occurs under reducing conditions, providing new insights into the cycling of arsenic in mixed phases of iron-rich, anoxic environments.

## Introduction

Arsenic (As) is a hazardous pollutant that significantly threatens human and environmental health worldwide. In several areas in the Global South, the As levels in soil and groundwater far exceed the current WHO standard of 10 μg L^–1^ due to natural geological processes and human activities such as mining, irrigation, and industrial operations.^1−5^ High As concentrations in various environments lead to its accumulation in plants and subsequent entry into the food chain.^6−8^

In environmental settings, As primarily exists in two oxidation states: As(III) and As(V). The redox transition between these states significantly influences the mobility, toxicity, and bioavailability of As.^3,9^ Factors such as pH, oxygen availability, and the presence of minerals, microbes, and organic substances play a crucial role in this redox transition.^10,11^ While the interactions of arsenic with iron (Fe) (oxyhydr)oxide minerals in soils and waters have been extensively studied,^6,12−14^ the role of mixed-valent Fe (oxyhydr)oxide minerals in As uptake remains underexplored.

It has been traditionally assumed that As(III) is more stable under reducing conditions, commonly found in anoxic environments such as groundwater and sediments. However, recent studies^15−17^ have shown that this assumption may be oversimplified. Emerging evidence^18−20^ suggests that certain Fe-bearing minerals, even under presumably reducing conditions, can unexpectedly promote the oxidation of As(III) to As(V). For example, Amstaetter et al. demonstrated that Fe(II) interacting with goethite could oxidize As(III) despite reductive conditions.^15^ Similarly, Ilgen et al. observed As(III) oxidation in the presence of reduced nontronite, an Fe-bearing clay mineral.^16,17^ These studies imply that Fe(II) acts as a catalyst for cryptic As(III) oxidation under reducing conditions. However, whether the cryptic As(III) oxidation occurs on mixed-valent Fe (oxyhydr)oxides, especially in the presence of organic ligands, remains unknown to date.

In this study, we investigate the oxidation of As(III) under pH-neutral anoxic conditions using green rust sulfate (GR-SO_4_), a mineral containing both Fe(II) and Fe(III) in its structure commonly found in oxygen-depleted soils.^21−23^ GR-SO_4_ has been found to effectively remove As from water,^26,27^ making it a relevant choice for our study. Additionally, we examine the impact of citrate, a naturally occurring organic ligand known for its dual role as a reductant and a stable Fe(III)-complexing agent, on the As(III) oxidation process. By conducting batch sorption experiments, spectroscopic analyses, and surface characterization techniques, we aim to elucidate the underlying mechanisms driving the unexpected As(III) oxidation in environments characterized by the coexistence of mixed-valent Fe minerals and organic ligands.

## Methods and Materials

### Chemicals and Solutions

All chemicals used were of analytical grade and are listed in Supporting Information (SI) Table S1. Aqueous solutions were prepared using high-purity deionized water (Purite, 18 MΩ cm) and deoxygenated by N_2_ gas purging before transferring to a glovebox (Coy Vinyl Anaerobic Chamber; ∼98% N_2_ and ∼2% H_2_).

### GR-SO_4_ Synthesis and Characterization

GR-SO_4_ was synthesized via a coprecipitation method^24,25^ under controlled anoxic conditions, as detailed in SI. Briefly, 50 mL of 0.3 M (NH_4_)_2_Fe(SO_4_)_2_·6H_2_O and 50 mL of 0.1 M FeSO_4_·7H_2_O were mixed with 100 mL of 0.3 M NaOH (final pH 8.0). After 1 h of aging, the suspension was centrifuged, washed, and dried under N_2_. Characterization using X-ray diffraction (XRD) and Transmission Electron Microscopy (TEM) confirmed phase purity (SI Figures S1 and S2). The ferrozine assay confirmed a 2:1 ratio of Fe(II) to Fe(III). Specific surface area was 42.9 m^2^ g^–^^1^ (BET method).

### As(III) Treatment with GR-SO_4_ with and without Organic Ligands

Batch experiments were conducted to examine the sorption of varied concentrations of As(III) (0, 50, 100, 250, and 500 μg L^–1^) onto GR-SO_4_ (1 g L^–1^; [Fe]_total_ ∼ 7.7 mM) inside a glovebox to ensure anoxic conditions (see details in SI). Parallel experiments with citrate (50 μM) or EDTA (200 μM) were also conducted. The experiments were conducted at pH 7.0 ± 0.1, using a fixed electrolyte of 0.01 M NaCl and 0.01 M HEPES buffer mixed to attain a neutral pH. All reactors were filled to equal volumes with deionized water, sealed, and crimped before being removed from the glovebox. The reactors were then placed on a rolling shaker to mix for 48 h in the dark to allow for equilibration. After 48 h, samples were taken from each reactor for subsequent analysis. ICP-OES (Agilent 5110) measurements were conducted to quantify the concentrations of As and Fe in filtered samples.

### X-ray Absorption Spectroscopy (XAS)

XAS analysis was conducted at the beamline BM28 of the European Synchrotron Radiation Facility (ESRF, Grenoble, France) on samples with 500 μg L^–1^ As(III) sorbed onto 1 g L^–1^ GR-SO_4_, with and without citrate. X-ray absorption near-edge structure (XANES) spectra were recorded at the As K-edge (11867 eV) and Fe K-edge (7112 eV) under anoxic conditions (more details in SI). Data were processed and analyzed using Athena^26^ (0.9.26), including linear combination fittings (LCF) to quantify As speciation changes and mineral phase transformations.

### Electron Microscopy

Samples, before (GR-SO_4_ both at pH 8.0 and pH 7.0) and after exposure to various As(III) treatments at pH 7.0, were prepared by centrifuging 1 mL of suspension. The resulting supernatant was discarded, and 200 μL of the sample was deposited onto a copper TEM grid coated with approximately 5 nm of graphite. Imaging was performed using a field-emission gun JEM-2100F from JEOL at 200 kV. Additionally, energy dispersive X-ray spectroscopy data was collected in Scanning TEM mode using an X-Max 80 mm^2^ EDX detector and analyzed using Aztec software, both from Oxford Instruments.

## Results and Discussion

### As(III) Sorption

**Figure 1:**
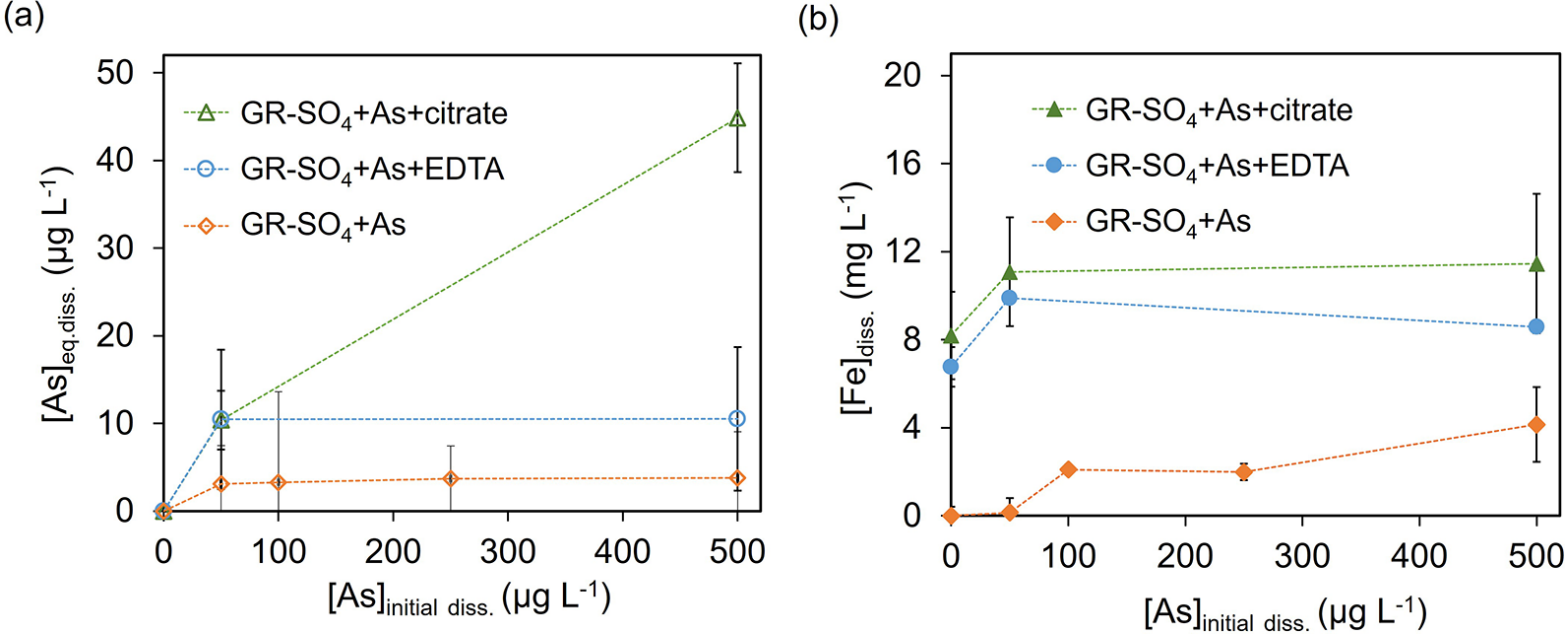
As(III) interaction with GR-SO_4_ at neutral pH in the presence and absence of citrate or EDTA. (a) equilibrium dissolved concentration of As (left panel; *Y*-axis, unfilled symbols) and (b) dissolved concentration of Fe (right panel, *Y*-axis, filled symbols) as a function of initial As concentration added as As(III) (*X*-axis). Batch sorption experiments were conducted with the 1 g L^–1^ suspension of GR-SO_4_ exposed for 48 h to varied initial concentrations of As(III) without ligands (symbol: diamonds) and with citrate (50 μM; triangles) or EDTA (200 μM; circles). All experiments were conducted under anoxic conditions. Error bars correspond to the standard deviations of triplicate measurements. The dotted lines serve as a visual guide.

A series of batch sorption experiments were carried out to investigate the interaction of As(III) with GR-SO_4_ (a suspension of 1 g L^–1^) under anoxic conditions at pH 7.0, both in the presence and absence of organic ligands. Results revealed that for GR-SO_4_+As, there was almost no dissolved As (maximum 3.8 μg L^–1^) detected across all As(III) concentrations, indicating near-total adsorption of As onto GR-SO_4_ (Figure 1a, unfilled diamonds). This highlights the high affinity and sorption capacity of GR-SO_4_ for the given As(III) concentration range, aligning with existing literature.^25^ The complete sorption of all As onto the GR-SO_4_ surface signifies a highly efficient process for removing As(III) from aqueous solutions (see SI, Figure S3).

The addition of citrate (50 μM) or EDTA (200 μM) increases the [As]_eq. diss._, compared to GR-SO_4_+As (Figure 1a). For GR-SO_4_+As+citrate, a slight rise in dissolved As (45 μg L^–1^) was observed for the highest initial As(III) concentration. At pH 7.0, citrate can serve as both a reductant and Fe-complexing agent,^27,28^ potentially leading to a decrease in available surface sites, thereby lowering the removal capacity at high concentrations of As(III). In contrast, for GR-SO_4_+As+EDTA, EDTA acts as a slow dissolution-promoting ligand at pH 7.0,^29^ resulting in less pronounced changes in dissolved As concentrations compared to GR-SO_4_+As+citrate.

In these experiments, the sorption followed the linearized form, equivalent to a log-transformed equation to the Freundlich equation, as described in Figure S3. This finding suggests that organic ligands may slightly inhibit As(III) removal from aqueous solutions at higher concentrations, possibly due to competitive sorption or complexation effects.

### Fe in Solution

We monitored [Fe]_diss._ to follow GR-SO_4_ dissolution during the experiments (Figure 1b). For GR-SO_4_+As, low concentrations of dissolved Fe were detected, which increased up to ∼0.4% of the total Fe in the system. The addition of ligands led to a release of Fe at ∼1.5% (see Figure S4). Previous studies have reported that citrate and EDTA lead to slow Fe (oxyhydr)oxide minerals dissolution at neutral pH, explaining our observations.^28−32^

## Arsenic Speciation

### XANES Analysis at As K-Edge

**Figure 2:**
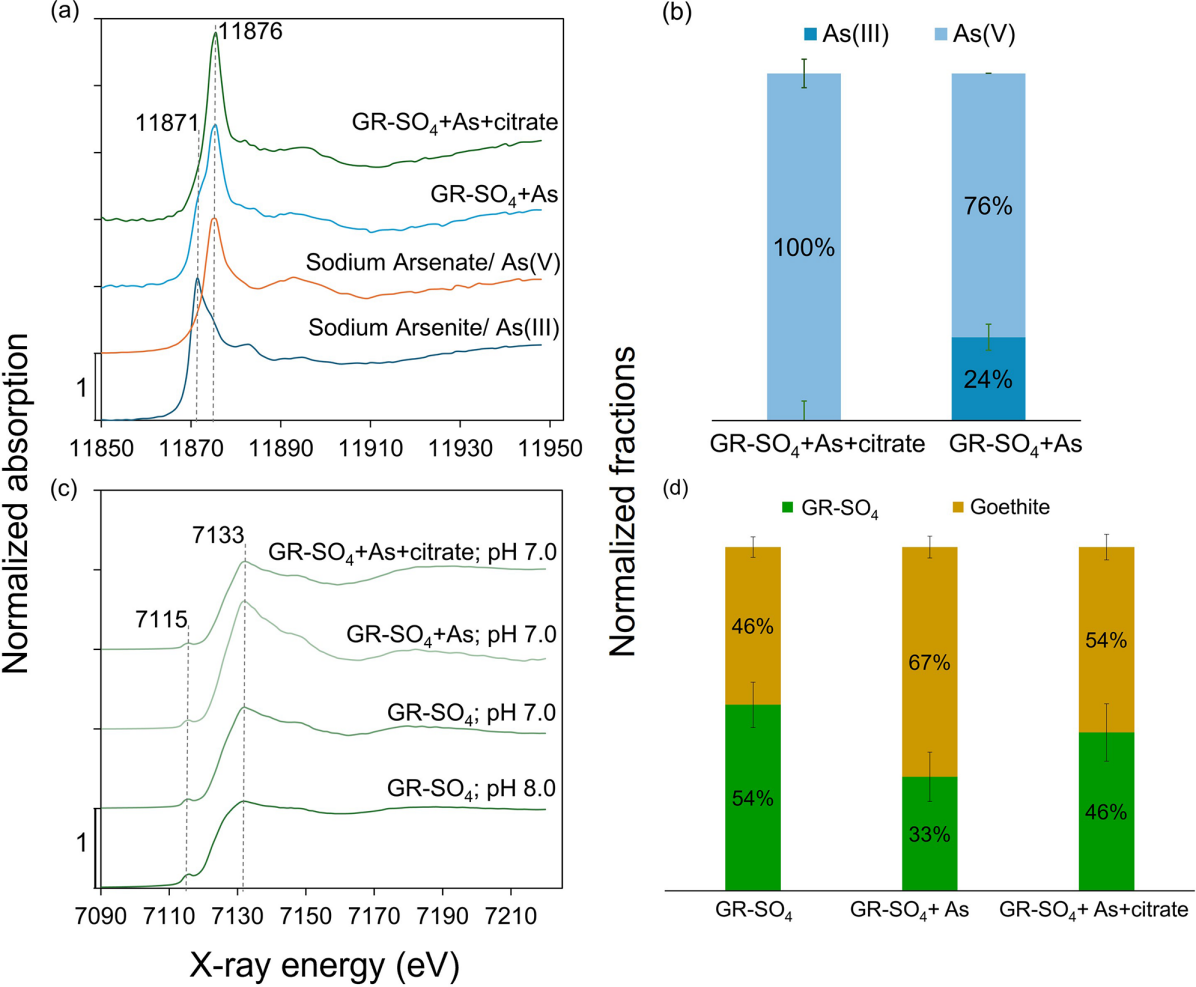
X-ray absorption near-edge structure (XANES) spectroscopy at As K-edge (a) and Fe K-edge (c) of solid samples collected before and after As(III) sorption experiments. The 1 g L^–1^ GR-SO_4_ suspension was exposed to As(III) (500 μg L^–1^) in the presence and absence of citrate (50 μM) at pH 7.0 under anoxic conditions. Calculated Liner Combination Fitting (LCF) fractions of XANES data presented for As speciation (2b) and different Fe phases (2d). Error estimates are shown in Table S2. The fitted fractions were normalized to a sum of 100%. XANES spectra of sodium arsenite and sodium arsenate for As speciation, and GR-SO_4_ synthesized at pH 8.0 (pure phase) and goethite for phase transformation were served as references amongst several other references (not shown) to fit the sample data.

We performed XANES spectroscopy at As K-edge to examine whether As speciation changes on the mineral surface. Figure 2a presents the XANES spectra for samples GR-SO_4_+As and GR-SO_4_+As+citrate and, for reference, sodium arsenite and sodium arsenate. The XANES spectrum of sodium arsenite (dark blue colored) displays a distinct absorption edge at 11871 eV, characteristic of As(III). In contrast, the spectrum for sodium arsenate (orange colored) reveals an absorption edge at 11876 eV, characteristic of As(V).

For the GR-SO_4_+As (light blue colored) sample, the absorption edge closely aligns with that of sodium arsenate at around 11876 eV. The spectrum also has a shoulder feature at 11871 eV that matches the As(III) reference. This spectrum suggests that As associated with GR-SO_4_ predominantly exists in the As(V) oxidation state, indicating effective oxidation of most of the added As(III) to As(V) but with some remaining As(III). In contrast, the GR-SO_4_+As+citrate (green colored) sample shows only an absorption edge at 11876 eV, indicating the presence of As(V) only.

LCF analysis indicates a substantial conversion of As(III) to As(V) by GR-SO_4,_ as seen in Figure 2b. In the GR-SO_4_+As sample, approximately 76% of the sorbed As(III) was oxidized to As(V), suggesting that the mineral phase had a significant but incomplete oxidative capacity under the experimental conditions. The partial oxidation is likely due to the presence of redox-active Fe(III) sites, which facilitate the oxidation of As(III) to As(V). However, the incomplete oxidation indicates a limitation in the oxidative capacity of the mineral phase, possibly due to the stabilization of Fe(II) within the green rust lattice, which could hinder further oxidation.

In the GR-SO_4_+As+citrate samples, the LCF analysis shows complete oxidation of As(III). The presence of citrate promotes complete As(III) oxidation, potentially owing to its complexation and electron-shuttling properties.

## Phase Transformation of GR-SO_4_

GR-SO_4_ was chemically synthesized according to established protocols at pH 8.0. Before any sorption experiments, the pH was lowered to 7.0_._ This change in pH was found to impact the stability of GR-SO_4_, which is consistent with previous research.^22,33−40^ We examined the solids using complementary techniques, XANES spectroscopy at Fe K-edge and TEM, to better understand phase transformation dynamics applicable to this study.

### XANES Analysis at Fe K-Edge

Figure 2c presents the normalized Fe K-edge XANES spectra for before- and after-sorption of As. The spectra show a distinct pre-edge feature around 7115 eV, characteristic of Fe(II) and Fe(III) in mixed valence states. The intensity and shape of the main absorption edge (7133 eV) and the postedge features differ among the samples, indicating variations in the Fe(II)/Fe(III) ratio and coordination environments, possibly leading to phase transformations.

The XANES spectrum of the GR-SO_4_ synthesized at pH 8.0 displays distinct features characteristic of pure GR-SO_4_; TEM and XRD analysis confirmed the purity of this sample. The absorption edge energy and pre-edge features indicate the presence of both Fe(II) and Fe(III) in the structure.

When the pH was decreased to 7.0, the spectrum of GR-SO_4_ highlighted significant changes. LCF analysis showed approximately 46% of GR-SO_4_ transformed into goethite (see Figure 2d). Similarly, the spectrum of the GR-SO_4_+As sample showed slight changes in the Fe oxidation state. LCF analysis of these samples indicated a predominance of goethite (67%) with a minor fraction of GR-SO_4_ (33%). The XANES spectrum of the GR-SO_4_+As+citrate sample also exhibited pronounced changes in the postedge region. LCF analysis revealed that 54% of GR-SO_4_ was transformed to goethite.

### TEM Analysis

**Figure 3:**
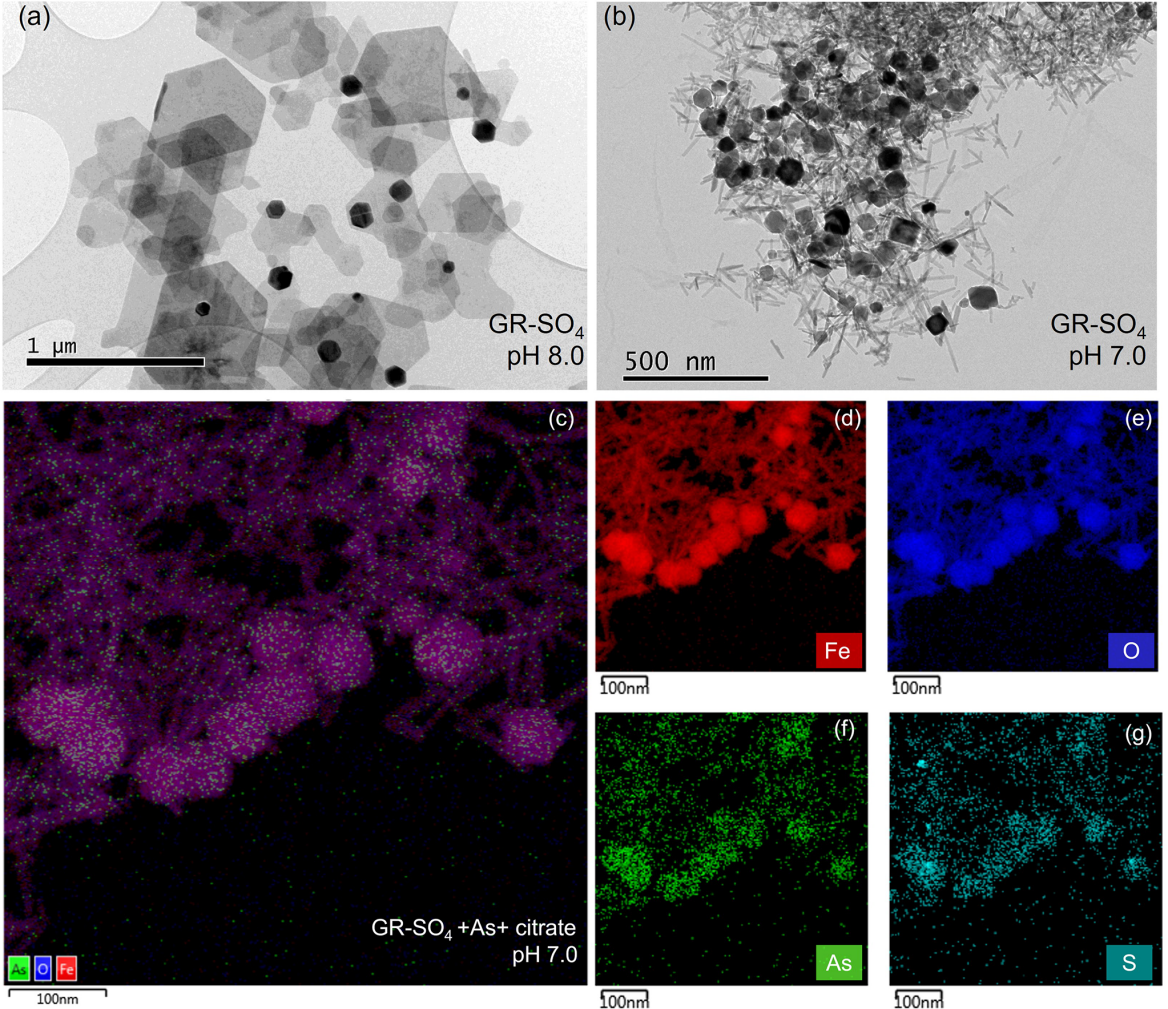
Transmission Electron Microscopy (TEM) and Energy Dispersive X-ray Spectroscopy (EDX) mapping of GR-SO_4_ and associated elements before and after sorption experiments. The micrographs of (a) GR-SO_4_ synthesized at pH 8.0, (b) GR-SO_4_ at pH 7.0, and (c-g) EDX images of the GR-SO_4_+As+citrate sample. The 1 g L^–1^ GR-SO_4_ suspension was exposed to As(III) (500 μg L^–1^) in the presence of citrate (50 μM) at pH 7.0 under anoxic conditions. The composite image (c) and individual element maps (d-g) show the distribution of Fe (red), O (blue), As (green), and S (teal).

Figure 3 presents the micrographs and EDX mapping of samples before and after sorption. The micrograph (Figure 3a) of the pure GR-SO_4_, which was synthesized at pH 8.0, presented a well-defined hexagonal plate-like morphology characteristic of GR-SO_4_.^40^ On the contrary, when pH was decreased to 7.0, the morphology of GR-SO_4_ was changed to a mix of needle or rod-like structures with hexagonal plates (Figure 3b), confirming that the mineral underwent phase transformation as earlier described by XANES analysis.

The EDX analysis of the GR-SO_4_+As+citrate sample provided spatially resolved elemental maps showing the distribution of Fe, O, S, and As on the mineral surface. The composite EDX image, as seen in Figure 3c, combines the individual element maps of Fe (red), O (blue), and As (green), demonstrating the colocalization of these elements. The overlap of Fe and As signals suggests that As adsorption occurs primarily in the Fe-rich areas of GR-SO_4_.

The individual EDX maps further clarify this distribution. The Fe map (Figure 3d) reveals a dense yet mixed-shaped morphology of Fe throughout the sample, consistent with the presence of GR-SO_4_ and goethite, as confirmed in Figure 2c-d. The O map (Figure 3e) shows the distribution of O, correlating closely with the Fe map, as expected due to the composition of GR-SO_4_ and goethite.

Figure 3f displays the localization of As, highlighting areas where As has been adsorbed onto the mixed mineral phase. Even after GR-SO_4_ transformation, the distinct presence of As in the EDX maps demonstrated that the sorption sites remained active and capable of binding As, which were initially added to the system as As(III). The As map qualitatively indicated a higher As concentration on the GR-SO_4_ surface than gothite, which is subject to future investigation.

## Mechanisms

### Phase Transformation and Mixed Mineral Phase Formation

GR-SO_4_ was synthesized at pH 8.0 with the Fe(II) to Fe(III) ratio of 2.0 in the solid. Upon lowering the pH from 8.0 to 7.0, the Fe(II) to Fe(III) ratio decreased from 2.0 to 0.38. This change might indicate at first that 81% of the initial Fe(II) was lost, oxidized, or transformed. This estimate would hold valid only if the Fe(III) concentration in the solid remained constant with an increased dissolved concentration of Fe, which was not the case as we observed goethite formation. Therefore, accounting for the loss of a maximum of 1.5% of total Fe in solution and assuming Fe(II) was oxidized to Fe(III), the initial Fe(II) underwent oxidation was estimated to be approximately 59% and thus generating new reactive Fe(III) species. Although we estimated the electron mass balance (see SI Table S3) using the available experimental parameters and observed results, we recognize that these are only approximations and do not capture the full complexity of the electron balance.

The pH change from 8.0 to 7.0 can induce changes in the local coordination environment and increase H^+^ on surfaces, destabilizing the interlayer and facilitating the transfer of electrons within the Fe(II)/Fe(III) in GR-SO_4_. This intramolecular electron transfer, accompanied by a protonation mechanism, results in electron delocalization and, ultimately, Fe(II) oxidation.

GR-SO_4_ has a reported pH_pzc_ within the range of 7.5 to 8.5.^41,42^ This means that under the experimental conditions, GR-SO_4_ carries a positive surface charge that could affect As(III) sorption. Previous studies have indicated that As(III) sorption onto pure GR-SO_4_ occurs within this pH range through inner-sphere complex formation with surface Fe.^25,43^ However, sorption mechanisms on mixed phases of Fe minerals may vary, as discussed below.

### As(III) Oxidation under Reducing Conditions

We have considered several potential mechanisms, as detailed in SI, to explain the cryptic As(III) oxidation.^15−17,20,47^ Mainly under the experimental conditions, the phase transformation of GR-SO_4_ into goethite generates reactive Fe(III) species capable of oxidizing As(III). As this transformation occurs, these newly formed labile Fe(III) can undergo a proton-coupled electron transfer, potentially producing transient Fe(IV) species. These highly reactive Fe(IV) intermediates could also oxidize As(III) even under anoxic, reducing conditions, as suggested by Hua et al.^46^

As(III) oxidation is more likely to occur at Fe(II)–O–Fe(III) sites than at Fe(III)–O–Fe(III) sites because the Fe(III)–O–Fe(III) bonds are stronger, making electron transfer less favorable, than Fe(II)–O–Fe(III).^29,30,44^ Excess electrons produced in the system could migrate into the interlayers of GR-SO_4_, potentially facilitating sulfate reduction. Reduced sulfur species might also contribute to As(III) oxidation,^45^ though their characterization was beyond the scope of this study.

### Role of Citrate in Enhancing Oxidation

Citrate, a naturally occurring organic ligand, enhances As(III) oxidation by strongly complexing with Fe, stabilizing it on the surface of GR-SO_4_/goethite. The low Fe dissolution observed in the presence of citrate (less than 2% over 48 h, as shown in Figure 1b and Figure S4) indicates that citrate effectively stabilizes Fe(III), preventing its immediate reduction back to Fe(II), thereby increasing the availability of Fe(III) for As(III) oxidation.

## Environmental Implications

Green rust commonly forms in natural environments with limited oxygen, such as waterlogged soils, wetlands, and aquifers. Numerous geogenic regions in South and Southeast Asia,^48−54^ including the Ganga-Brahmaputra-Meghna Delta, Mekong Delta, and Red River Delta, exhibit Fe(III)-reducing conditions and redox cycling, which significantly affect As mobility. Our study can elucidate the presence of As(V) in some regions of the Holocene floodplains,^54^ despite conditions that theoretically favor As(III).

The presence of organic ligands such as citrate, commonly found in natural environments due to root exudates and microbial activity, increases the oxidative capacity of mixed phases of Fe minerals,^28,32^ enhancing the oxidation of As(III) to As(V) and thus leading to As retention. Additionally, the role of sulfur in As immobilization, as observed in peatlands^45,55^ and peat-containing aquifers,^56^ where reduced sulfur facilitates As(III) oxidation, might be similar to the processes observed with GR-SO_4_ in our study.

Green rust minerals intercalate various anions such as sulfate, carbonate, and chloride into their layered structures, which significantly affects their reactivity and their ability to trap contaminants.^23,33,35,57^ In this study, the phase transformation of GR-SO_4_ into secondary Fe phases like goethite promotes the oxidation of As(III) and the retention of As(V), providing potential pathways to immobilize As. These processes are consistent with studies on zerovalent iron (ZVI) based water treatment systems,^58,59^ where the cyclic transformations of Fe phases, including lepidocrocite and green rust carbonate, increase As retention.

Overall, the unexpected oxidation of As(III) under conditions typically considered reducing extends the understanding of As cycling in dynamic redox environments and highlights the complex interplay between Fe mineralogy, organic ligands, and As speciation in natural and engineered systems.

## Supporting Information Available

List of chemicals used in this study; details on Methods and Materials; characterization of GR-SO_4_ using XRD and TEM; sorption of As(III) onto GR-SO_4_ in the presence and absence of citrate; dissolution of Fe minerals; LCF of XANES (derivatives) of As and Fe K-edge spectra; experimental parameters and estimates on excess electrons; As(III) oxidation mechansims (PDF)
Compilation of the presented experimental data (XLSX)
