# Supplementary material for: Redox Dynamic Interactions of Arsenic(III) with Green Rust Sulfate in the Presence of Citrate
Source: Environ Sci Technol Lett. 2024 Oct 15;11(11):1239–46. doi: 10.1021/acs.estlett.4c00700 (PMC11562726; doi:10.1021/acs.estlett.4c00700)
Supplement: Supplementary file 1 — ez4c00700_si_001.pdf [file ez4c00700_si_001.pdf]

Supporting Information

Redox Dynamic Interactions of Arsenic(III) with Green Rust Sulfate in the Presence of Citrate

Jagannath Biswakarma[1*], Molly Matthews[1], and James M. Byrne[1]

[1]School of Earth Sciences, University of Bristol, Bristol, BS8 1RJ, United Kingdom

[*]Email: jagannath.biswakarma@bristol.ac.uk; jagannathbiswakarma@gmail.com

Index

Table S1.	List of chemicals used in this study
Methods and Materials:	1. GR-SO_4 Synthesis and Characterization
	2. As(III) treatment with GR-SO_4 with and without organic ligands
	3. X-ray Absorption Spectroscopy
Figure S1.	Characterization of GR-SO_4 using XRD
Figure S2.	Characterization of GR-SO_4 using TEM
Figure S3.	As(III) sorption onto GR-SO_4 with and without citrate
Figure S4.	Dissolution of Fe
Table S2.	LCF of XANES (derivatives) of As and Fe K-edge spectra
Table S3.	Experimental parameters and estimate on excess electrons
Mechanisms:	As(III) oxidation under reducing conditions

Table S1. List of chemicals used in this study

**Chemical Name**	**Formulae**	**Supplier**	**Purity (%)**
Ammonium iron(II) sulfate	$(NH_4)_2Fe(SO_4)_2 \cdot 6H_2O$	Fisher Chemical	> 99
Iron(III) sulfate hydrate	$FeSO_4 \cdot 7H_2O$	Sigma Aldrich	97
Sodium hydroxide	NaOH	Fisher Chemical	99.1
Sodium chloride	NaCl	Fisher Chemical	> 99.5
HEPES buffer	$C_8H_{18}N_2O_4S$	Fisher Bioreagent	99
Green rust sulfate (GR-SO_4)	-	Lab-synthesized	-
Sodium Arsenite	$NaAsO_2$	Sigma Aldrich	> 98
Sodium Citrate	$C_6H_5Na_3O_7.2H_2O$	Merck	> 99
Ethylenediaminetetraacetic acid	$C_{10}H_{16}N_2O_8$	Sigma Aldrich	> 99

Methods and Materials

1. $GR\text{-}SO_4$ Synthesis and Characterization

$GR\text{-}SO_4$ was synthesized via a co-precipitation method[1, 2] under controlled anoxic conditions inside a glovebox. Here, 50 mL of 0.3 M $(NH_4)_2Fe(SO_4)_2{\cdot}6H_2O$ and 50 mL of 0.1 M $FeSO_4{\cdot}7H_2O$ were mixed in a 300 mL PFA reactor. To this mixture, 100 mL of 0.3 M NaOH was added dropwise, ensuring the final pH reached approximately 8.0. Continuous stirring and pH monitoring were employed while adding NaOH to ensure uniform precipitation. The resultant dark blue-green suspension was aged for one hour, then centrifuged at 4000 rpm for 30 minutes to separate the solid phase. The precipitated solid was thoroughly washed with deionized deoxygenated water to eliminate impurities. The washed $GR\text{-}SO_4$ was then dried under a nitrogen atmosphere to prevent oxidation and stored in sealed containers within the glovebox. Characterization of the synthesized mineral using X-ray diffraction (XRD) and Transmission Electron Microscopy (TEM) confirmed phase purity, see SI Figures S1 and S2. The ferrozine assay confirmed a 2:1 ratio of Fe(II) to Fe(III). The specific surface area was measured as 42.9 $m^2\ g^{-1}$ by a multipoint N_2 Brunauer Emmett Teller (BET) adsorption method (Anton Paar Autosorb iQ).

2. As(III) treatment with $GR\text{-}SO_4$ with and without organic ligands

Batch experiments were conducted at neutral pH to examine As(III) sorption onto $GR\text{-}SO_4$ in the presence and absence of organic ligands, mainly citrate. Ethylenediamine tetraacetic acid (EDTA) was also employed to examine sorption in batch experiments. A concentration of 1 g L^{-1} of freshly precipitated $GR\text{-}SO_4$ suspension (total [Fe] ~ 7.7 mM) was prepared in an anoxic environment to prevent oxidation of surface and bulk Fe(II). The experiments were conducted at pH 7.0±0.1, using a fixed electrolyte of 0.01 M NaCl and a buffer of 0.01 M HEPES mixed to attain neutral pH. Reactors were set up inside a glovebox to ensure anoxic conditions for two sets of treatments. In one set ($GR\text{-}SO_4$+As), only As(III) was added at five environmentally relevant concentrations: 0, 50, 100, 250, and 500 μg L^{-1} into the buffered mineral suspension. In another set of experiments ($GR\text{-}SO_4$+As+citrate or $GR\text{-}SO_4$+As+EDTA), 50 μM citrate (or 200 μM EDTA) was added concurrently with As(III) at 0, 50, and 500 μg L^{-1} concentrations to examine the effects of organic ligands on As(III) sorption onto the mineral surface. All reactors were filled to equal volumes with deionized water, sealed, and crimped before being removed from the glovebox. The reactors were then placed on a rolling shaker to mix for 48 hours to allow for equilibration. All experiments were conducted in triplicate. After the 48-hour equilibration period, 5 mL samples were taken from each reactor, centrifuged at 1000 rpm, and filtered through 0.20 μm non-pyrogenic sterile filters (Filtropur S 0.2). The supernatants were preserved with 5 mL of 1% HNO_3 for subsequent analysis by inductively coupled plasma optical emission spectrometry (ICP-OES; Agilent 5110), and solid fractions were stored under anoxic conditions for XAS and TEM analysis.

3. X-ray Absorption Spectroscopy (XAS)

XAS analysis was conducted at the beamline BM28 (XMaS) of the European Synchrotron Radiation Facility (ESRF) in Grenoble, France. This analysis was performed on samples with 500 μg L^{-1} As(III) sorbed onto 1 g L^{-1} suspension of GR-SO_4 under anoxic conditions in the presence and absence of 50 μM citrate at pH 7.0. Samples were made from the solid fraction collected after each sorption experiment. Each sample was sealed between Kapton tapes inside the glovebox and mounted into the XAS sample holder. X-ray absorption near-edge structure (XANES) spectra were recorded at As K-edge (11867 eV) and Fe K-edge (7112 eV) under anoxic N_2 environments in fluorescence and/or transmission detection mode, depending on the sample. Multiple (4-10) scans were recorded for each sample depending on the data quality and to improve the signal-to-noise ratio. All scans of each sample were carefully checked for any beam-induced changes, which were not observed. Data were processed and analysed with Athena (0.9.26).[3] Spectra of each measured sample were merged, energy shift corrected, and E0 set by selecting the maximum intensity of the first derivative. Merged spectra were normalized using the Autoback routine of Athena. Many references consisting of As(III), As(V), and pure phases of iron minerals were also measured. Linear combination fittings (LCF) of As and Fe K-edge XANES data were performed with the derivatives by using the respective functions of Athena in order to quantify the As speciation changes and mineral phase transformations.

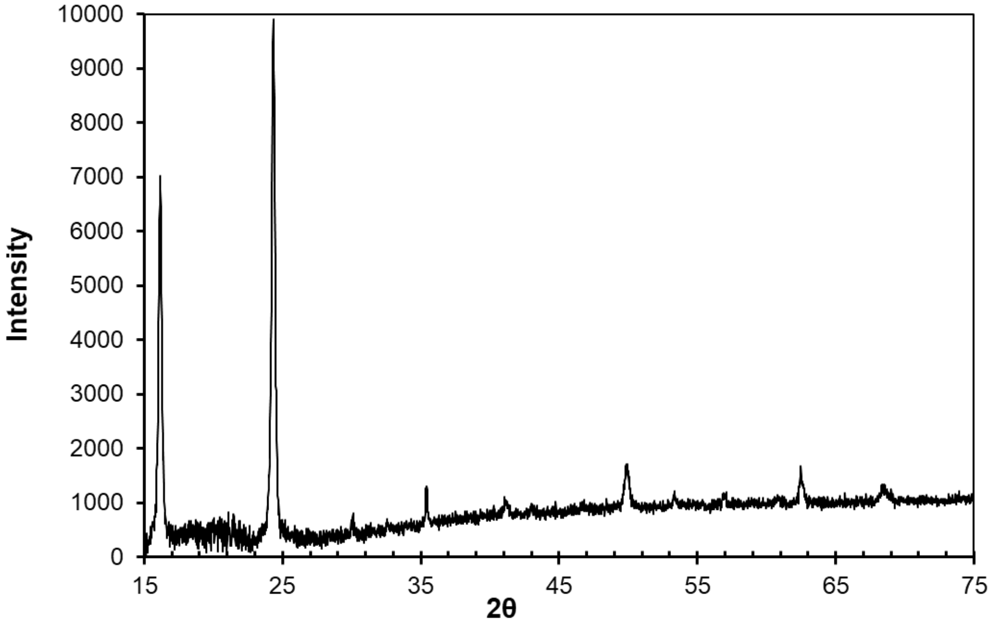

Figure S1. Characterization of GR-SO_4 synthesized at pH 8.0 using X-ray diffraction. XRD measurements were performed using a Bruker D8 Advance with a PSD LynxEye detector and Cu radiation (1.5406 Å). Samples were put onto low background silicon wafer holders, and data was collected using the following parameters: angle range 15-75°, step size 0.02°, and one second dwell time. Samples were compared against a database to confirm the purity of the mineral phase.

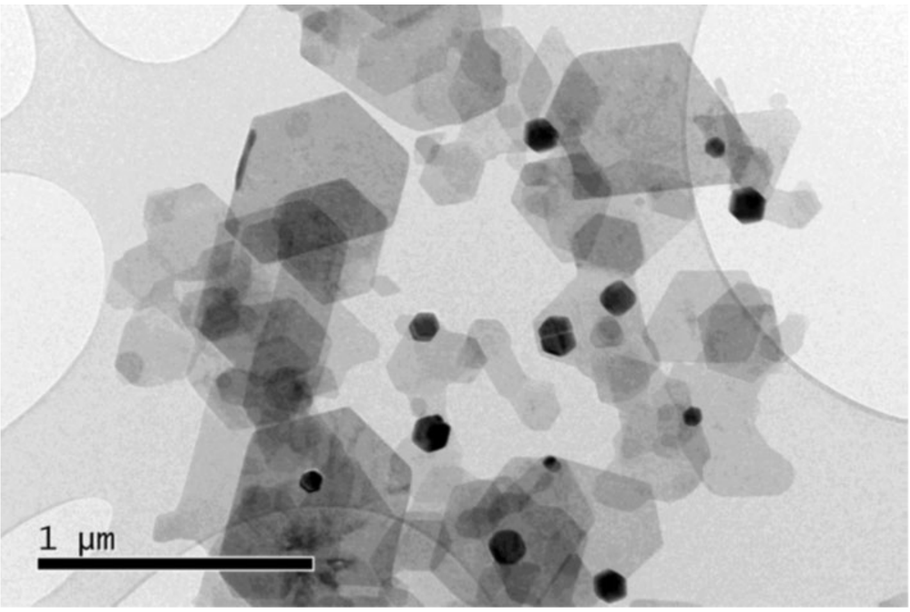

Figure S2. Characterization of GR-SO_4 synthesized at pH 8.0 using TEM (exact figure also shown in Figure 3a. Please read the main text in the manuscript for more details).

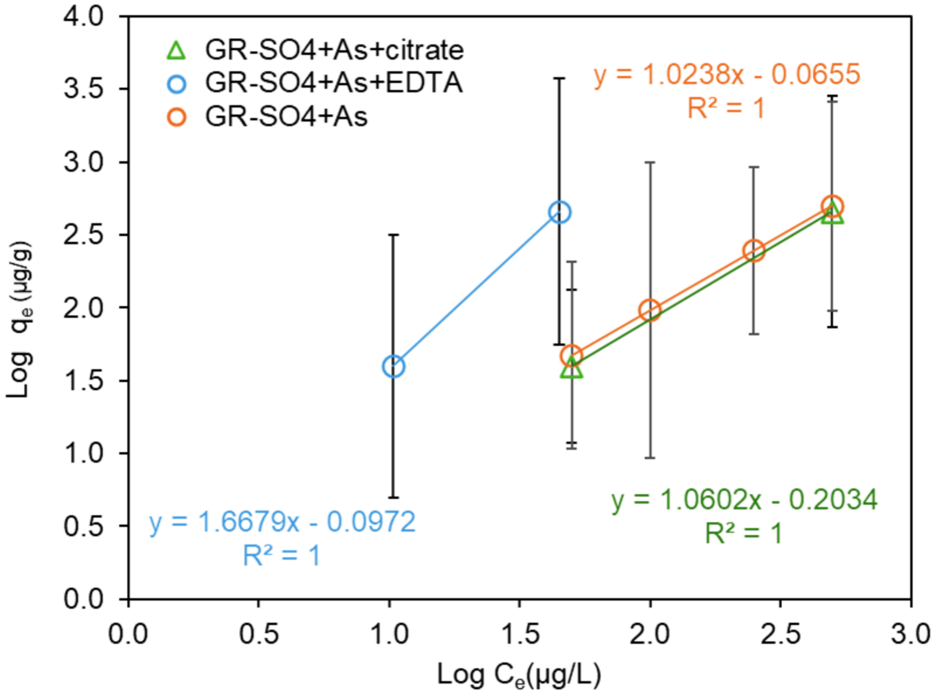

Figure S3. As(III) sorption onto GR-SO_4 at pH 7.0 under anoxic conditions. This figure presents the logarithmic values of the sorbed fraction of As (Log q_e, µg g^{-1}) against the logarithmic values of the dissolved equilibrium concentration of As (Log C_e, µg L^{-1}). A linear regression line (equivalent to the Log linearised form of the Freundlich equation) was used to fit the data.

$$Log(q_e) = Log(K_F) + \frac{1}{n} Log(C_e)$$

where:

q_e is the amount of adsorbate adsorbed per unit mass of adsorbent (µg g^{-1}),

C_e is the equilibrium concentration of the adsorbate in the solution (µg L^{-1}),

K_F is the Freundlich adsorption coefficient, indicating the capacity of the adsorbent

1/n is a dimensionless constant related to the intensity of the adsorption process.

From this equation, we extracted sorption parameters K_F and 1/n, which are expressed in equations in the figure (colors: orange for GR-SO_4+As, green for GR-SO_4+As+citrate, blue for GR-SO_4+As+EDTA).

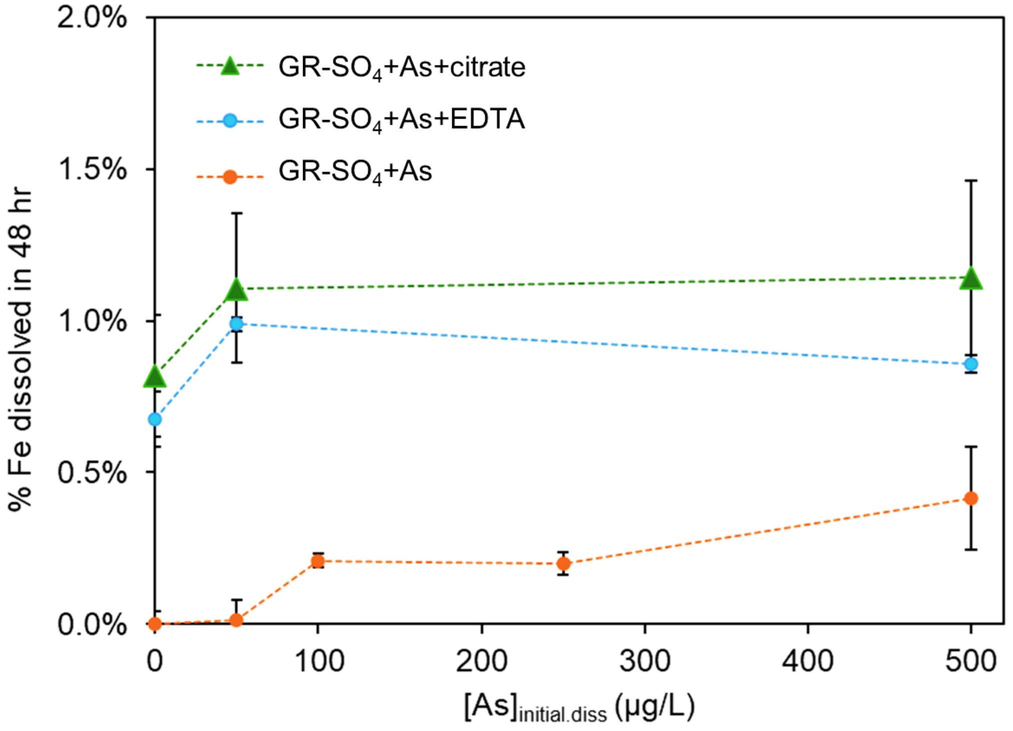

Figure S4: Dissolution of iron minerals, expressed in % Fe dissolved after 48 hr. Dissolution was monitored before and 48 hr after As(III) addition. Varied concentrations of As(III) were added into the GR-SO_4 (1 g L^{-1}) suspension in the presence and absence of citrate (50 µM) or EDTA (200 µM). All experiments were conducted at pH 7.0 under anoxic conditions.

Table S2. LCF-derived fractions of XANES (derivatives) of As (a) and Fe (b) K-edge spectra. Fractions normalized over the sum of the fitted fractions.

(a) As K-edge

**Samples**	**As(III)**	**As(V)**	**rfactor**	**chinu**	**chisqr**
GR-SO_4+As	0.24	0.76	0.1156	0.0026	0.1099
GR-SO_4+As+citrate	0	1	0.1524	0.0059	0.2533

(b) Fe K-edge

**Samples**	**Pure GR-SO_4**	**Goethite**	**rfactor**	**chinu**	**chisqr**
GR-SO_4; pH 7.0	0.54	0.46	0.0227	0.0001	0.0026
GR-SO_4+As	0.33	0.67	0.0389	0.0002	0.0080
GR-SO_4+As+citrate	0.46	0.54	0.0409	0.0001	0.0037

Table S3. Experimental parameters and estimate on excess electrons

Samples	Conditions	Value	Comments
$GR-SO_4$ (pH 8.0)	[$GR-SO_4$]	1 g L^{-1}	Suspension concentration for our experiments
	$[Fe]_{total}$	7.7 mM	Total Fe in solid ($GR-SO_4$)
	Fe(II)/Fe(III) ratio	2	Initial Fe(II)/Fe(III) ratio in $GR-SO_4$; measured with Ferrozine assay
	[Fe(II)]	5.1 mM	initial Fe(II) concentration
	[Fe(III)]	2.6 mM	initial Fe(III) concentration
Post pH change:			
$GR-SO_4$ (pH 7.0)	Fe(II)/Fe(III) ratio	0.38	After pH adjustment to 7.0
	$[Fe]_{total}$	7.58 mM	Accounting for a maximum of 1.5% dissolved Fe
	[Fe(II)]	2.10 mM	After pH adjustment to 7.0
	[Fe(III)]	5.48 mM	After pH adjustment to 7.0
	$GR-SO_4$ transformation	46% to goethite	Fraction of $GR-SO_4$ transformed to goethite; XANES Fe K-edge analysis
	Remaining $GR-SO_4$	54%	Fraction of $GR-SO_4$ in the solid
	Moles of Fe(II) oxidized	3.0 mM	Moles of Fe(II) oxidized during pH adjustment
	Electrons from Fe(II) oxidation	3.0 mmol e^-	Electrons released by Fe(II) oxidation
	Electrons balance	3.0 mmol e^-	Total electrons available before As(III) addition
Post As(III) addition ($GR-SO_4$ +As(III) at pH 7.0)			
	[As(III)]	500 µg L^{-1}	Concentration of As(III) added
		~ 6.68 µM	Total moles of As(III)
	As(III) oxidized	76%	% of As(III) oxidation; XANES
		~ 5.07 µM	Moles of As(III) oxidized to As(V)
	Electrons from As(III) oxidation	10.1 µmol e^-	Electrons released from As(III) oxidation

Mechanisms: As(III) oxidation under reducing conditions

In our study, we have considered several potential mechanisms to explain the cryptic As(III) oxidation consistent with recent studies.[4-8]

A. One plausible mechanism for the observed As(III) oxidation is due to the catalytic role of Fe(II) available in GR-SO_4, complementary to some recent studies.[4, 7] This process involves the following steps: *1. Adsorption of As(III) on GR-SO_4 surface:* As(III) in solution can sorb onto the positively charged GR-SO_4 surface at neutral pH. The adsorbed As(III) is in close proximity to the Fe(II) and Fe(III) species present on the mineral surface, creating a microenvironment conducive to redox reactions. *2. Electron transfer from Fe(II) to Fe(III):* The presence of Fe(II) within the GR-SO_4 structure or on its surface can transfer electrons to Fe(III) sites, reducing them to Fe(II) and generating reactive Fe(III) species. These new labile Fe(III) sites are highly oxidizing and can interact with As(III). *3. Oxidation of As(III) by reactive Fe(III) species:* The reactive Fe(III) species generated at the GR-SO_4 surface act as oxidants, converting sorbed As(III) to As(V). *4. Role of organic ligands in enhancing oxidation:* Citrate, a naturally occurring organic ligand, is known for its strong complexation with Fe(III). When citrate is present, it can complex with Fe(III) species, stabilizing them in solution and surface. The low Fe dissolution in the presence of citrate (less than 2% in 48 hours, as shown in Figure 1b and Figure S4) suggests that citrate effectively stabilizes Fe(III) on the surface, facilitating As(III) oxidation without significant loss of Fe to the solution. This stabilization mainly prevents the immediate reduction of Fe(III) back to Fe(II), thereby increasing the availability of Fe(III) for As(III) oxidation. Additionally, citrate may influence the structure and reactivity of GR-SO_4, potentially facilitating its transformation into more oxidizing goethite, as seen in Figure 2d. These newly formed phases can further contribute to the complete As(III) oxidation, and thus, the addition of citrate can enhance the oxidation of As(III) to As(V). The role of EDTA on As speciation remained open for future research.

B. An alternate mechanism would involve the direct participation of already available Fe(III) in the mixed GR-SO_4-goethite to act as an electron acceptor, causing As(III) to transfer electrons to Fe(III), thereby reducing Fe(III) to Fe(II) and oxidizing As(III) to As(V). However, this mechanism is unlikely to occur where only Fe(III)-O-Fe(III) moieties are dominant because usually the bonds between Fe(III)-O-Fe(III) are stronger than Fe(II)-O-Fe(III)[9-11], to participate in redox reactions. It is possible that due to the high specific surface area of GR-SO_4, As(III) sorption might occur preferentially onto Fe(II)/Fe(III) sites of GR-SO_4 compared to goethite, as seen in Figure 3c, but further investigation is needed to confirm this mechanism.

C. Under the experimental conditions, the phase transformation of GR-SO_4 at neutral pH into a more stable goethite phase could contribute to the observed As(III) oxidation. This transformation could generate new reactive Fe(III) species that are particularly effective in oxidizing As(III). Excess electrons in the system may migrate into the interlayers of GR-SO_4, potentially facilitating sulfate reduction. Reduced sulfur species could also contribute to the oxidation of As(III).[12] However, quantifying and characterizing the sulfur species involved in this process was beyond the scope of our investigation.

D. Our findings hint that interfacial electron exchange during phase transformation could form reactive Fe(IV) intermediates. In this process, Fe(II) transfers electrons to the Fe(III) within GR-SO_4, converting Fe(II) into labile Fe(III). This newly formed Fe(III) can then undergo a proton-coupled electron transfer, generating transient Fe(IV) species. These highly reactive Fe(IV) intermediates can oxidize reductive species like As(III), as suggested by Hua et al.[13], offering a mechanistic explanation for As(III) oxidation in our system under anoxic conditions.

While speculative, all these proposed mechanisms align well with the observations of cryptic As(III) oxidation in other iron-rich systems[4-8] and best explain our observations.

References

(1) Géhin, A.; Ruby, C.; Abdelmoula, M.; Benali, O.; Ghanbaja, J.; Refait, P.; Génin, J. M. R. Synthesis of Fe(II-III) Hydroxysulphate Green Rust by Coprecipitation. *Solid State Sciences* **2002**, *4* (1), 61-66, DOI: 10.1016/S1293-2558(01)01219-5

(2) Perez, J. P. H.; Freeman, H. M.; Schuessler, J. A.; Benning, L. G. The Interfacial Reactivity of Arsenic Species with Green Rust Sulfate ($GR_{(SO4)}$). *Sci Total Environ* **2019**, *648*, 1161-1170, DOI: 10.1016/j.scitotenv.2018.08.163

(3) Ravel, B.; Newville, M. ATHENA, ARTEMIS, HEPHAESTUS: Data Analysis for X-Ray Absorption Spectroscopy Using IFEFFIT. *J Synchrotron Radiat* **2005**, *12*, 537-541, DOI: 10.1107/S0909049505012719

(4) Amstaetter, K.; Borch, T.; Larese-Casanova, P.; Kappler, A. Redox Transformation of Arsenic by Fe(Ii)-Activated Goethite (α-FeOOH). *Environ Sci Technol* **2010**, *44* (1), 102-108, DOI: 10.1021/es901274s

(5) Ilgen, A. G.; Kruichak, J. N.; Artyushkova, K.; Newville, M. G.; Sun, C. J. Redox Transformations of As and Se at the Surfaces of Natural and Synthetic Ferric Nontronites: Role of Structural and Adsorbed Fe(II). *Environ Sci Technol* **2017**, *51* (19), 11105-11114, DOI: 10.1021/acs.est.7b03058

(6) Ilgen, A. G.; Kukkadapu, R. K.; Leung, K.; Washington, R. E. "Switching on" Iron in Clay Minerals. *Environ Sci-Nano* **2019**, *6* (6), 1704-1715, DOI: 10.1039/c9en00228f

(7) Zhang, X.; Fu, Q.; Hu, H.; Zhu, J.; Fang, L. Iron Minerals Enhance Fe(II)-Mediated Abiotic As(III) Oxidation. *Chemosphere* **2024**, *363*, 142913, DOI: 10.1016/j.chemosphere.2024.142913

(8) Zhao, Z. W.; Meng, Y.; Wang, Y. H.; Lin, L. M.; Xie, F. Y.; Luan, F. B. Anaerobic Oxidation of Arsenite by Bioreduced Nontronite. *J Environ Sci* **2021**, *110*, 21-27, DOI: 10.1016/j.jes.2021.03.011

(9) Biswakarma, J.; Kang, K.; Borowski, S. C.; Schenkeveld, W. D. C.; Kraemer, S. M.; Hering, J. G.; Hug, S. J. Fe(II)-Catalyzed Ligand-Controlled Dissolution of Iron(Hydr)Oxides. *Environ Sci Technol* **2019**, *53* (1), 88-97, DOI: 10.1021/acs.est.8b03910

(10) Biswakarma, J.; Kang, K.; Schenkeveld, W. D. C.; Kraemer, S. M.; Hering, J. G.; Hug, S. J. Linking Isotope Exchange with Fe(II)-Catalyzed Dissolution of Iron(Hydr)Oxides in the Presence of the Bacterial Siderophore Desferrioxamine-B. *Environ Sci Technol* **2020**, *54* (2), 768-777, DOI: 10.1021/acs.est.9b04235

(11) Biswakarma, J.; Kang, K.; Schenkeveld, W. D. C.; Kraemer, S. M.; Hering, J. G.; Hug, S. J. Catalytic Effects of Photogenerated Fe(II) on the Ligand-Controlled Dissolution of Iron(Hydr)Oxides by EDTA and DFOB. *Chemosphere* **2021**, *263*, 128188, DOI: 10.1016/j.chemosphere.2020.128188

(12) Besold, J.; Biswas, A.; Suess, E.; Scheinost, A. C.; Rossberg, A.; Mikutta, C.; Kretzschmar, R.; Gustafsson, J. P.; Planer-Friedrich, B. Monothioarsenate Transformation Kinetics Determining Arsenic Sequestration by Sulfhydryl Groups of Peat. *Environ Sci Technol* **2018**, *52* (13), 7317-7326, DOI: 10.1021/acs.est.8b01542

(13) Hua, J.; Fei, Y. H.; Feng, C. H.; Liu, C. S.; Liang, S.; Wang, S. L.; Wu, F. Anoxic Oxidation of As(III) During Fe(II)-Induced Goethite Recrystallization: Evidence and Importance of Fe(IV) Intermediate. *J Hazard Mater* **2022**, *421*, DOI: 10.1016/j.jhazmat.2021.126806
